# A supervisory controller intended to arrest dynamic falls with a wearable cold-gas thruster

**DOI:** 10.1017/wtc.2023.18

**Published:** 2023-09-06

**Authors:** Almaskhan Baimyshev, Michael Finn-Henry, Michael Goldfarb

**Affiliations:** School of Engineering, Vanderbilt University, Nashville, TN, USA

**Keywords:** design, exosuits, feedback devices, human–robot interaction, mechatronics

## Abstract

This article examines the feasibility of employing a cold-gas thruster (CGT), intended as a backpack-wearable device, for purposes of arresting backward falls, and in particular describes a supervisory controller that, for some motion described by an arbitrary combination of center-of-mass angle and angular velocity, both detects an impending fall and determines when to initiate thrust in the CGT in order to arrest the impending fall. The CGT prototype and the supervisory controller are described and experimentally assessed using a rocking block apparatus intended to approximate a backward-falling human. In these experiments, the CGT and supervisory controller restored upright stability to the rocking block in all experiment cases that would have otherwise resulted in a fall without the CGT assistance. Since the controller and experiments employ a reduced-order model of a falling human, the authors also conducted a series of simulations intended to examine the extent to which the controller might remain effective in the case of a multi-segment human. The results of these simulations suggest that the CGT controller would be nearly as effective on a multi-segment falling human as on the reduced-order model.

## 1. Introduction

Falls are one of the leading causes of injury and injury-related death for the elderly (Bergen et al., 2016). In addition, falls can lead to fear of falling and subsequent loss of independence (Ambrose et al., 2013). With age, balance control systems deteriorate both in the sensory (visual, vestibular, and proprioceptive) and actuation (strength, agility, and reaction time) aspects (Lockhart et al., 2005). To reduce the likelihood of falling, several fall prevention measures are available for home environments, such as handrails, higher friction flooring, and improved lighting. However, most falls occur outside such controlled spaces (Lord et al., 1993); as such, there is a need for portable fall prevention devices for use outside home.

Several researchers have investigated the use of lower-limb exoskeletal devices to facilitate balance, which could potentially preclude the initiation of falls (e.g., Vallery et al., 2012; Tucker et al., 2015; Ugurlu et al., 2015; Wang et al., 2015; Yan et al., 2015; Di et al., 2016; Huynh et al., 2018; Zhang et al., 2018; Fasola et al., 2019; Hamza et al., 2020). Employing a lower limb exoskeleton to arrest the onset of an initiated fall, however, has not yet been studied. Using a lower-limb exoskeleton to arrest an impending fall would entail assessment of the dynamic configuration of the falling user, computation of an appropriate corrective action, and high-bandwidth closed-loop actuation to realize this action. Since the exoskeleton intervention is applied to the limbs of the wearer, the recovery strategy of the exoskeleton must coordinate with the wearer’s own efforts (i.e., limb movements) at balance recovery, which substantially complicates the control problem.

Providing assistance to arrest an impending fall is presumably simpler if a device can apply a corrective force or moment directly to the user relative to the inertial reference frame (IRF), rather than a floor-referenced force or moment through the legs, since the need to coordinate exoskeleton leg movement with user leg movement is lessened. One means of providing assistance relative to the IRF is via a control moment gyroscope (CMG). These devices can be worn as a backpack and assist the wearer to maintain balance by applying restorative moments in a closed-loop manner (Li and Vallery, 2012; Matsuzaki and Fujimoto, 2013; Chiu and Goswami, 2014; Lemus et al., 2017; Oya and Fujimoto, 2017). A recent study of one such device indicated that a CMG greatly improved balance of human users with relatively low-magnitude assistive moments (Lemus et al., 2020).

An alternative means to apply a corrective force directly to the user relative to the IRF is via a thruster. Accordingly, the authors have previously conducted preliminary investigations into the feasibility of employing a backpack-wearable cold-gas thruster (CGT) for purposes of arresting backward falls (Finn-Henry et al., 2020; Baimyshev et al., 2022). The CGT is intended as a backpack-worn safety device for individuals at fall risk, intended to arrest a fall in progress. The device is intended to provide for a single fall intervention per charge of gas (i.e., requires recharging prior to subsequent use). Such use is consistent with the expected low frequency of falling in elderly individuals; namely, of falls reported among the elderly, approximately half fell once; one quarter fell twice; and one quarter fell three or more times over a 1-year period (Florence et al., 2018). Although falls are infrequent, they comprise a substantial share of healthcare expenditures for adults over 65 years (Florence et al., 2018).

In the prior investigation of feasibility, the design of the CGT was presented, and the control authority of a prototype was experimentally characterized using a rocking block experimental apparatus to model a backward-falling human (Baimyshev et al., 2022). In that paper, the controller was shown to restore upright equilibrium to a rocking block that leaned at some initial angle with zero initial velocity. The present article continues the feasibility investigation of the CGT approach, and in particular, develops and experimentally assesses an autonomous supervisory controller that accommodates dynamic falls, and both: (1) detects a dynamic fall in progress, and also; (2) determines when the CGT should be energized, based on the estimated angle and angular velocity of the user’s center of mass with respect to their base of support. This supervisory autonomous controller is experimentally assessed on an updated version of the CGT prototype (relative to the previous publication), using an updated version of a rocking block apparatus (relative to the previous publication), which is intended to approximate a backward-falling human. A video of these experiments is included with the Supplementary Material to complement data presented in the article. Additionally, a series of simulations are presented which are intended to extrapolate the experimental results on the rocking block to the expected multi-link characteristics of a human body – specifically, a three-segment model with ankle and hip joints, with biomimetic torque limits. Therefore, the main contribution of this work is the development of an autonomous supervisory controller that determines when to energize the CGT as a function of center-of-mass angle and angular velocity to restore upright balance while being as conservative as possible.

## 2. CGT prototype

**Figure 1.**
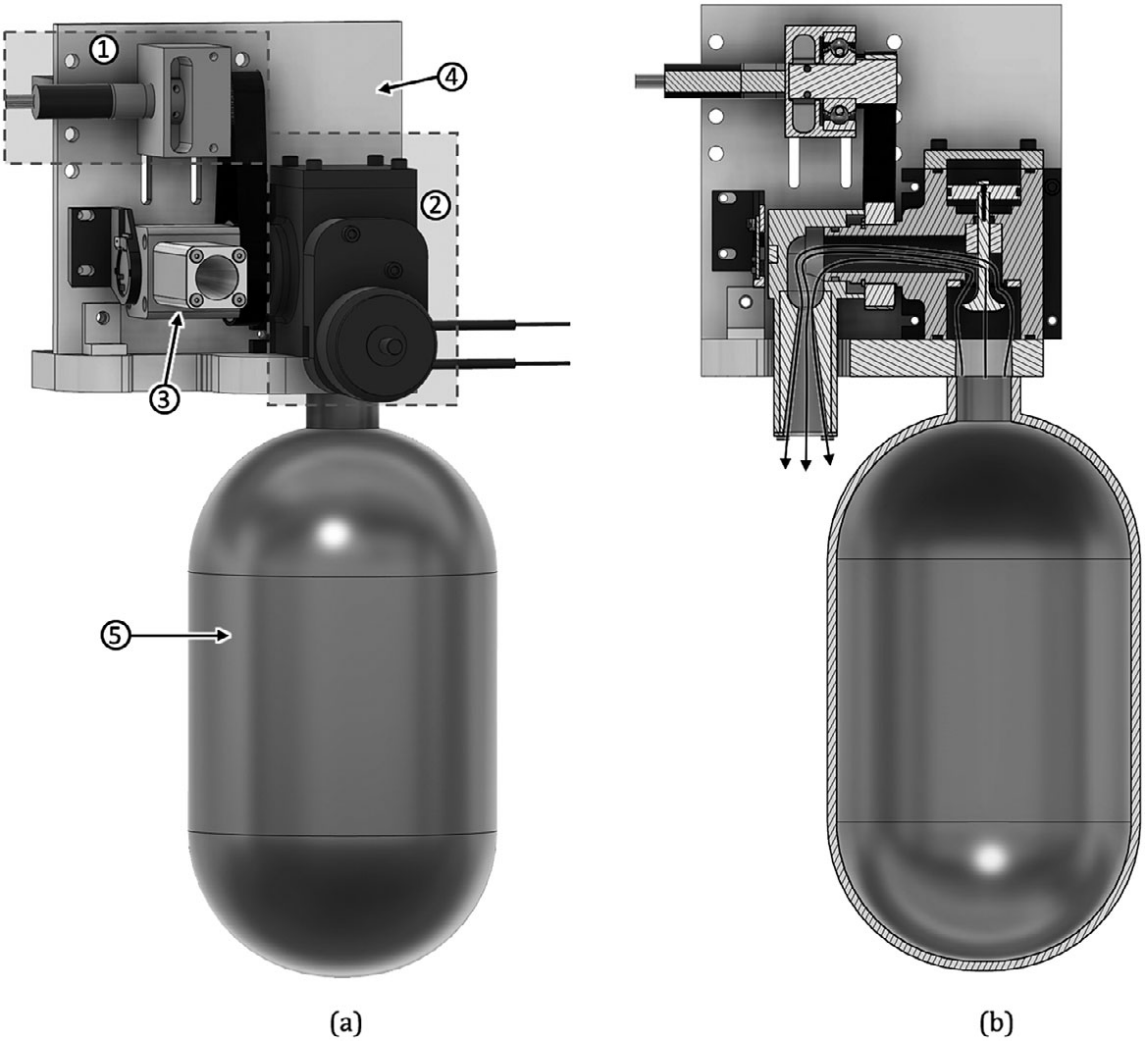
(a) CGT prototype (shown without refilling valves). The labels identify (1) servo system, (2) custom piloted poppet valve, (3) thruster nozzle, (4) mounting plate, and (5) carbon fiber tank; (b) CGT prototype in cross-section showing the flow path of nitrogen gas when the CGT is triggered. Note that the nozzle is shown in-plane in order to illustrate the cold gas flow path.

The CGT prototype and its constituent components are shown in a solid model in Figure 1. The design of the CGT prototype was described in detail in Baimyshev et al. (2022), and is described here only briefly for completeness. The version presented in this article is a slightly modified version of the design presented in Baimyshev et al. (2022), with updates to the solenoid-actuated valve and nozzle servo system to improve reliability and functionality, although otherwise is functionally very similar to the previous prototype. The CGT cross-section shown in Figure 1b illustrates the path of the cold gas flow from the compressed gas tank, through the (normally closed) solenoid-actuated valve (shown with valve open), and then out through the nozzle to create the restorative thrust force (note that the nozzle is shown in Figure 1b in-plane to illustrate more clearly the gas flow path). The carbon-fiber compressed gas tank has a volume of 1 L and is initially charged with nitrogen gas to a pressure of 10 MPa (1,500 psi). When energized by the controller, the solenoid opens the normally closed poppet valve, which releases the compressed gas through the nozzle. The resulting thrust impulse provides a decaying thrust profile with an initial thrust of approximately 350 N, which decays to zero over a period of approximately 700 ms. See Baimyshev et al. (2022) for the specific thrust characteristics. The CGT also includes an on-board embedded system that employs a six-axis IMU for sensing, as well as control electronics for the servocontrol axis and actuation of the poppet valve. The assembled CGT prototype has a total mass of 2 kg and a volumetric envelope of 30 cm × 18 cm × 11 cm.

## 3. CGT supervisory controller

### 3.1. Considering backward falls

The focus of the present study is to explore the prospective utility of a CGT to arrest backward falls. Falls can occur in along three primary directions: forward, backward, and sideways. Although the proposed approach could be adapted to all three, the present feasibility study focuses on backward falls. A recent study of the community-dwelling elderly reported that backward falls comprise over 40% of total falls; 20% of backward falls resulted in injury; and approximately 10% resulted in an emergency department visit (Nevitt and Cummings, 1993; Crenshaw et al., 2017). A person falling backward has little visual feedback and least effective fall mitigation techniques (Tan et al., 2006). The limit of stability (LOS) in backward direction is the smallest, meaning there is little room for center of pressure (COP) excursion before a fall is initiated. In case of an impending fall, a person has two main strategies to prevent an impact with the ground. After an initial delay due to reaction time, a typical response is an attempt to step in the direction of the fall, also known as change-in-support strategy (Maki et al., 2003). The effectiveness of such strategy, however, is reduced in the backward direction due to the range of motion of leg joints, which results in the lowest rate of recovery from posterior perturbations, as noted by Hsiao and Robinovitch (1997). The second strategy is described by Suzuki et al. (2012) as the “stable anti-phase” hip strategy, where the trunk (and/or arms) moves out of phase with the legs to reduce the displacement of the COM. Depending on the velocity of the COM, these strategies may be sufficient to recover upright balance. Due to changes in the visual, vestibular, and musculoskeletal systems associated with older age, however, elderly people are less successful in utilizing these strategies (King et al., 1994). Finally, despite the fact that sideway falls result in a higher hip fracture rate (Parkkari et al., 1999), assistive devices for balance assistance, such as a rolling walker, are least effective in the backward direction. Therefore, in the case of backward falls: (1) limits of stability (LOS) are the smallest; (2) visual feedback is most limited; (3) mitigation techniques are least effective; and (4) existing assistive devices (e.g., a rolling walker) have limited utility. As such, for the purposes of this feasibility study, this article considers the objective of arresting a backward fall. If a CGT can be successfully implemented with this functionality, it can presumably be expanded in future work to consider other fall directions.

### 3.2. Control overview

The CGT is intended as an interventional device that detects when a fall is occurring and applies a corrective force to arrest (or counteract) the fall, in order to aid the user in regaining balance – much the same way another person might intervene in the event of an impending fall. Rather than return the user to a singular equilibrium point, the control objective is to assist the user in returning to his or her basin of stability (from which he or she would presumably regain balance). This basin of stability approach is similar to some previously proposed approaches for the analysis of upright balance that also employ phase plane analyses, such as the capture point (e.g., Pratt et al., 2006) and extrapolated center of mass (e.g., Hof et al., 2005; Hof, 2008) methods of analysis. Similar methods were also experimentally explored in human subject studies of balance recovery by Pai and Patton (1997), Patton et al. (1999), and Simoneau and Corbeil (2005).

Further, rather than attempt to modulate the magnitude of the thrust during the thrust intervention, the CGT delivers the full thrust impulse at an appropriate time, with the intent of arresting the impending fall and returning the user to a state from which he or she can recover balance. Note that controlling thrust in real-time would require a thrust control system with considerable authority and bandwidth, which is likely not viable for a backpack-worn device, particularly since the thrust event only lasts roughly 0.7 s. Therefore, the essence of the control approach is to calculate the appropriate time to apply a thrust impulse of known magnitude in order to arrest an impending fall and return the user to sufficiently upright configuration, from which the user can regain balance.

Given this approach, the authors seek a controller that waits as long as possible to provide corrective assistance, allowing the user to recover without CGT assistance if at all possible. Doing so saves the thrust intervention for when it is absolutely necessary, and also minimizes the likelihood of a false positive (i.e., incorrectly identifying a fall). As such, the proposed intervention is used somewhat like an airbag might be, although it is used early in the fall to help the user regain upright standing balance, rather than late in the fall to mitigate impact. Given this general approach to the problem, the control objective requires a controller that detects a (backward) fall in progress and determines the latest moment (within a design margin) for the onset of CGT thrust that will return the user to a state from which he or she is likely to recover.

### 3.3. Control approach

A number of fall detection approaches have been proposed in the engineering literature, primarily focused on the elderly population. Various proposed approaches employ wearable sensors, such as accelerometers and gyroscopes, and some approaches employ external or ambient visual sensors, such as RGB and/or depth cameras with feature tracking or the combination of the two, sometimes augmented with vital signal sensors (Chen et al., 2005; Noury et al., 2007; Kangas et al., 2008; Mubashir et al., 2013; Planinc and Kampel, 2013; de Miguel et al., 2017; Dedabrishvili et al., 2021). The main objective of fall detection systems in these works is predominantly to detect when a fall has occurred (i.e., when a person has made contact with the ground). As such, the two main sources of information for wearable sensors are the fall impact, which produces a distinguishable spike in acceleration signals, and the horizontal position of the body for an extended period after the fall. For purposes of initiating a corrective action from the CGT, such detection would be too late, since the aim of the CGT is to detect and correct a fall in progress, and therefore to preclude the impact event. As such, the impending fall must be detected well before impact, and also well before there is a large deviation from the upright posture. Nyan et al. (2008) propose a pre-impact fall detection for use with inflatable hip protectors, although the detection occurs past the limits of control authority of the proposed device (10° angle and around 90°/s angular velocity). Therefore, the CGT supervisory controller requires a method of fall prediction, rather than fall detection, in order to initiate a control action.

**Figure 2.**
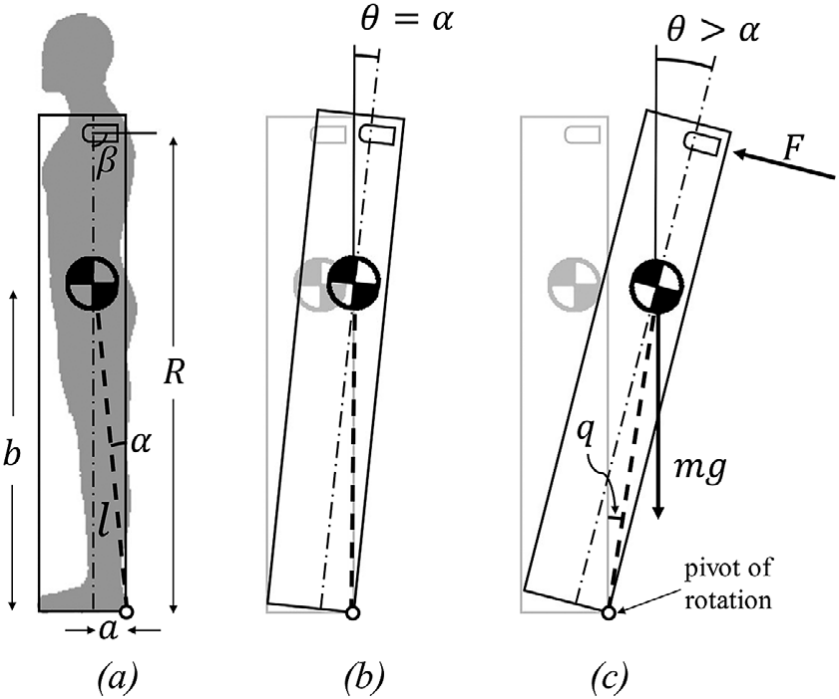
Rocking block when (a) stationary, (b) at fall angle, and (c) falling. Note that the control objective is based on the variable *q*, labeled in (c).

In order to predict impending falls, the CGT controller employs a simplified dynamic model of a backward-falling human, which is used to estimate when the modeled system will recover from a perturbation, and when it will not. Specifically, the backward-falling human is approximated as a rocking block, as described in Baimyshev et al. (2022) and shown in Figure 2. A rocking block, unlike an inverted pendulum, is characterized by a region of stability rather than a single point of equilibrium. When the rocking block is tilted such that the center of mass remains above its base of support and released from rest, the block will return to a stable equilibrium; when tilted such that the center of mass is no longer above the base of support and released from rest, the block will fall. The second-order nonlinear dynamics of a rocking block in a plane as a function of time *t* can be described by:
(1)

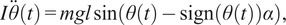

 where 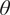 is the angle of the block with respect to the vertical, *I* and *m* are the moment of inertia around the pivot of rotation (at the heel of the falling person) and mass of the block respectively, *g* is the gravity constant, and 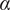 is the slenderness angle (calculated as 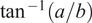 ) or the fall angle as shown in Figure 2. Note that the extent to which this simplified model approximates a backward-falling person is explored in Section 6. The basin of stability of (1) can be characterized in the 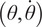 phase plane, where the boundary of stability is the locus of points outside of which the system will fall away from the origin, rather than converge toward it.

When supplemented by the CGT, the dynamics of the rocking block (1) are modified to include the CGT thrust force, which, as presented previously in Baimyshev et al. (2022), can be described as:
(2)

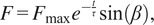

 where 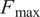 and 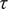 are the thrust amplitude and time constant, and 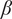 is the CGT nozzle angle with respect to the equivalent pendulum. The CGT-modified rocking block dynamics therefore become:
(3)

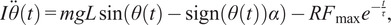

 where the thrust force is applied by the CGT to the block at height *R*, and it is further assumed the nozzle angle is 90°, for the purposes of the control approach. The basin of stability of the modified dynamics (3) can be characterized in the 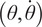 phase plane with a boundary of stability, defined by the locus of points outside of which the system will fall away from the origin, rather than converge toward it. This modified boundary represents the extent of the region in which triggering the CGT will reconfigure the rocking block back into the unmodified region of stability (i.e., it describes the boundary in phase space within which activating the CGT will return the system to a stable state). The CGT control law essentially employs a closed-form solution to (3) to identify in real-time the boundary of stability in the phase plane and activates the CGT as the rocking block approaches this (modified) boundary. In other words, the CGT control law is implemented such that the CGT is activated when the block states are at the upper boundary of the CGT-expanded stability basin. The controller continuously measures the current block angle and angular velocity, and for some current angle, calculates the maximum angular velocity of the block from which the applied thrust is capable of returning the block to upright equilibrium. Once the current angular velocity of the block is equal to the calculated maximum angular velocity, the CGT is activated.

The aim of the CGT is to configure the block to the angle of 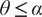 , after which the block can settle into an upright equilibrium after a period of rocking (i.e., after which the human user could presumably regain balance). Since the controller is only used in the region where the block accelerates toward a fall outside the LOS, that is, 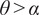 , the entity 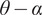 can be replaced by 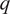 or the angle of the pendulum of length *L* with equivalent physical parameters of the block, pivoting around the contact point of the corner of the block with the ground (i.e., the equivalent of a user pivoting on his/her heels). The controller objective then becomes bringing the pendulum angle 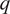 to zero. Thus, the equation of motion (3) can be rewritten with the new variable 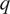 as:
(4)

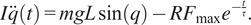



This equation of motion can be linearized around an operating point 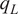 , such that:
(5)

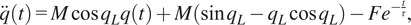

where 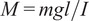 and 
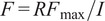
 . A closed-form solution of (5) can be obtained using a Laplace transformation, partial fractions, and inverse Laplace transformation, and can be described by:
(6)

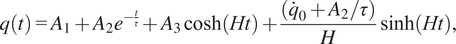

where 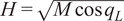 , 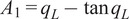 , 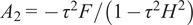 , and 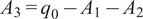 . Knowing the initial conditions 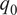 and 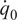 , (6) can be used to algebraically estimate the angle 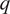 at some time *t.* Since this is a predictive controller, the “initial conditions” in the model, 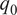 and 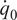 , correspond to the current state of the pendulum.

The closed-form solution for the motion of the pendulum following the thrust event can then be used to solve the following problem: for the given current angle 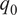 , find the angular velocity that will result in an angle 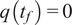 , where 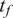 is the duration of the thrust (approximately 0.7 s for the CGT employed here). The corresponding angular velocity from which the CGT will return the pendulum to upright stability is:
(7)

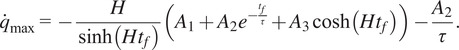



If the current measured angular velocity is equal to the calculated angular velocity (7) and the CGT is activated, the block should return to upright equilibrium. Hence, for a given pendulum angle 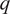 , the upper boundary of the CGT-expanded stability basin can be considered as the maximum angular velocity 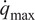 given by (7), since for any velocity greater than (7) the CGT would not have enough control authority to successfully return the block to equilibrium. As such, the supervisory controller continuously measures the states of the block and activates the CGT when the measured angular velocity 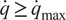 .

**Figure 3.**
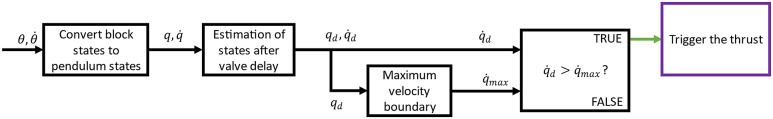
The control diagram of the CGT controller.

Finally, for purposes of implementation, the control law is modified slightly to account for the time delay in thrust generation (i.e., CGT thrust will not commence immediately, but rather entails a short delay, 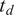 between commanding of the thrust force and generation of the thrust), the model is used to estimate future states. Therefore, instead of employing the current measured angle and angular velocity as input to (7), the controller instead uses (6) to estimate the future angle 
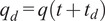
 , and differentiates (6) with respect to time to estimate the future angular velocity 
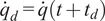
 , then uses the values 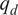 and 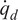 instead of the current measured angle and angular as input to (7). The control approach with future prediction is diagrammed in Figure 3.

## 4. Experimental assessment

### 4.1. Experimental apparatus

**Figure 4.**
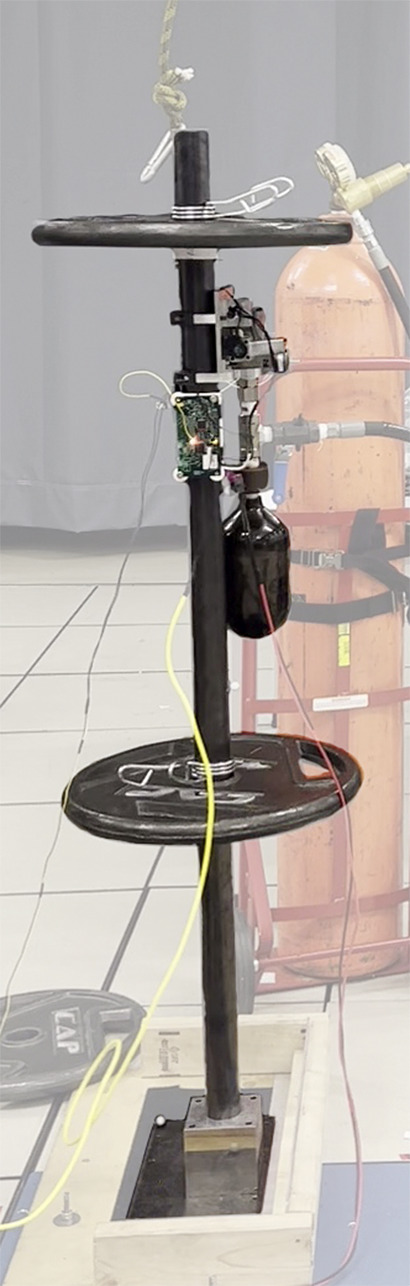
The rocking block experimental apparatus with the CGT prototype is attached. Note that the CGT prototype as shown includes a supplemental quick-connect fitting between the CGT compressed gas tank and a nitrogen refill tank, which facilitates refilling the CGT tank between successive experimental trials.

The CGT controller – described by equation (7) and Figure 3 – was implemented on the CGT prototype, and the combination was mounted to and tested on a rocking block experimental apparatus, as shown in Figure 4, where the parameters of the rocking block were selected to approximate the physical characteristics of a standing human. Note that the version of the CGT prototype shown in Figure 4 includes a supplemental quick-connect fitting introduced between the CGT compressed gas tank and a nitrogen refill tank, which was mounted as a temporary measure to facilitate refilling the CGT tank between successive experimental trials. Table 1 lists the parameters of the rocking apparatus, relative to those of a 1.55-m-tall person with a mass of 52 kg, where the inertial characteristics of the human were approximated using relationships given by Winter (2009), and the forward and backward LOS based on published data by Brouwer et al. (1998) and Shepard (2016). In order to emulate the ability of the human to regain balance once their COM is returned to a region in the phase space within the stability basin (of equation (1)), Velcro strips were attached at the interface of the base and floor, which provided the apparatus with an approximate coefficient of restitution (COR) of 0.7. The COR was determined by leaning the block to an angle within its LOS and letting it rock back and forth, recording the homogeneous angle response, then finding the best-fit COR by matching the response in simulation. Note that the COR of 0.7 was implemented to approximate the balance recovery dynamics observed in human balance recovery, as described in a previous paper (Baimyshev et al., 2022). Note that the extent to which the rocking block model approximates a backward-falling human is examined in Section 6.

**Table 1.** Comparison of physical parameters of constructed rocking blocks and a human body

	Human body	Experimental apparatus
Mass (kg)	52	52
Height (m)	1.55	–
COM height (m)	0.87	0.87
Moment of inertia (kg∙m^2^)	55.5	53
Forward LOS (deg)	5.6–8	8
Backward LOS, 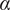 (deg)	2.9–4	4

### 4.2. Controller implementation

The CGT was affixed to the rocking block with the nozzle placed at approximately chest level, or specifically at 75% of the total height (see Figure 4). The nozzle angle was configured perpendicular to the pendular axis of the block, which provided maximum control authority. The CGT controller continuously measured the angle and the angular velocity of the block ( 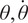 ) using the onboard IMU (TDK InvenSense MPU-6050), calculated equivalent pendulum states ( 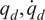 ) taking into account the valve delay, and performed the control computation described by equation (7), with 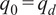 and 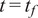 , as shown in Figure 3. Note that angle measurement employed a complementary filter-based sensor fusion approach, using the inverse tangent of the two sagittal-plane axes of the accelerometer for the low-frequency component and integration of the sagittal-plane gyroscope for the high-frequency component. All parameters associated with the controller implementation are given in Table 2, while the reasoning for selecting these parameters is explained in detail in Baimyshev et al. (2022). The measured states were then used to calculate the maximum allowable velocity 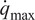 corresponding to the upper boundary of the CGT-expanded stability basin at 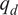 . The CGT remained idle until 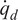 exceeded 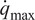 , at which point the controller triggered the assistive thrust. The control loop was implemented at a sampling frequency of 1 kHz on an embedded system (based around the Microchip DSPIC33FJ64GS608-50I/P microcontroller) that was affixed to the CGT.

**Table 2.** Controller parameters

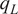	10°
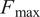	350 N
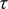	140 ms
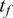	700 ms
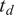	20 ms

### 4.3. Experiment protocol

**Figure 5.**
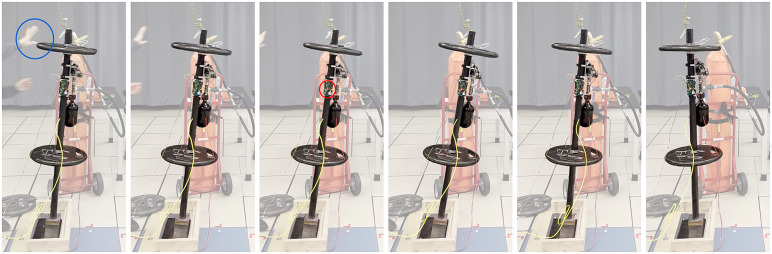
Frames from a video of a representative experiment with the CGT are attached. Frame 1: the researcher has finished pushing, the hand is not contacting the block (blue circle); 2: the block is falling; 3: the thrust has been triggered, onboard LED switched color from green to red (red circle); 4: the block angular velocity has been brought down to zero; 5: the block returning to its inherent stability basin; 6: upright equilibrium recovered. A video of the experiments is included with the Supplementary Material.

Experiments were conducted to assess the prospective efficacy of the proposed controller and CGT assistance in restoring stability to the rocking block apparatus. In these experiments, the block was pushed in the backward direction by a researcher, which imparted to it some initial angle and angular velocity, where the initial conditions were recorded immediately following release of the block. All angle and angular velocity data were measured using a Vicon motion capture system. Three reflective markers were attached to the base of the block to measure the tilt angle, and after each push, the angular motion of the block was recorded by a 12-camera motion capture system (Vicon, Oxford, GBR) at 200 Hz. Two conditions were tested: (1) without triggering the CGT thrust and (2) triggering the CGT thrust using the CGT controller previously described. For each case (with and without triggering CGT thrust), the block was pushed ten times, with the experimenter attempting to vary the phase space initial conditions. Note that a slack rope was attached to the block to catch it when the angle 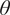 reached approximately 35°, in order to prevent subsequent impact with the floor. For the CGT thrust trials, the 1L CGT tank was refilled for each successive trial. A video camera, time-synchronized with the motion capture data, was also used to record each trial, specifically to ascertain when the experimenter released the block, and when the CGT was triggered. Note finally that the CGT controller utilized only measurement from the IMU, while the motion capture data was used to record the angular motion of the rocking block for each trial for subsequent analysis. During data processing, the RMS error between the IMU and the motion capture angle data was found to be 0.4°. Figure 5 shows a series of frames from the video camera illustrating a trial in which CGT thrust was triggered. The first frame indicates the moment at which the experimenter released the block (indicated by the blue circle), which corresponds to the initial condition of that trial. The third frame shows the moment at which the CGT controller triggered the CGT thrust, as signaled by an LED on the embedded system switching from green to red (indicated by the red circle in the figure). The remaining frames illustrate the “recovery” of the rocking block (i.e., the block returned to within its stability basin). Note that a video showing these experiments is included in the Supplementary Material.

## 5. Results

**Figure 6.**
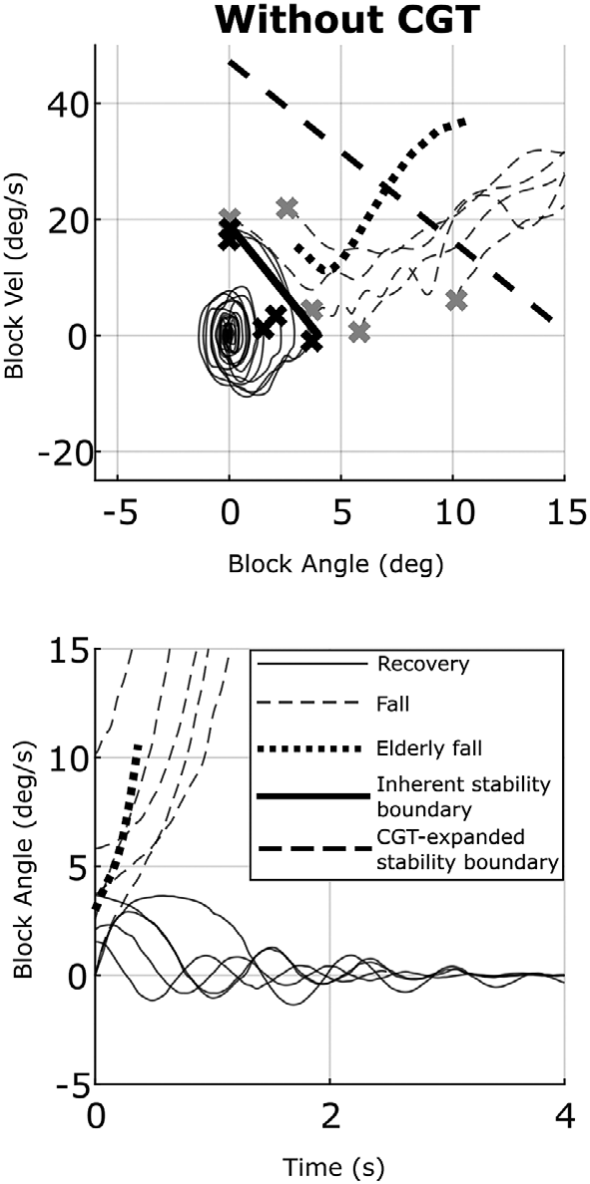
Phase plots of the block states (top row) and the block angle versus time (bottom row) for experiments without CGT assistance. Crosses represent the initial conditions of each experiment. The solid black line indicates the model-based limits of stability for the unassisted rocking block, while the dashed black line indicates the model-based limits of stability for the CGT-assisted block. The solid traces are the cases for which the rocking block recovered from the initial conditions, while the dashed traces are the cases for which the block fell. Note that the dotted line shows the fall dynamics of an elderly person extracted from a video.

**Figure 7.**
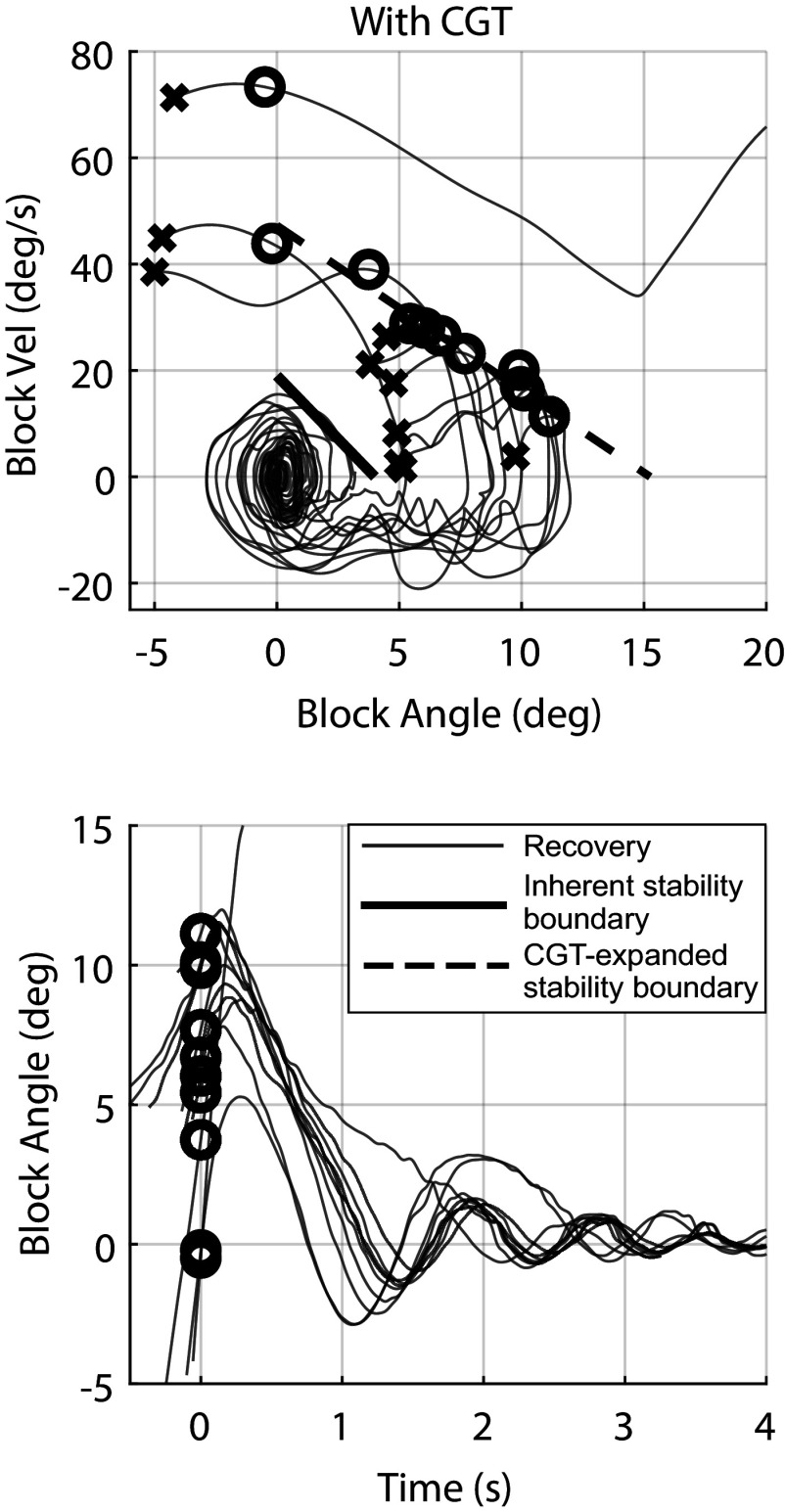
Phase plots of the block states (top row) and the block angle versus time (bottom row) for experiments with CGT assistance. Crosses represent the initial conditions of each experiment, while circles represent the instant at which thrust was triggered. The solid black line indicates the model-based limits of stability for the unassisted rocking block, while the dashed black line indicates the model-based limits of stability for the CGT-assisted block.

The results of the experiments, showing the response of the rocking block to being pushed, without CGT assistance and with it are shown in phase and time plots in Figures 6 and 7, respectively. Specifically, the phase plane in Figure 6 shows the angle and angular velocity trajectories corresponding to each trial without CGT assistance. The initial conditions corresponding to each trial are shown as *x*’s on the phase plot, indicating the initial angle and angular velocity of the block immediately after being released by the researcher. The limits of stability region from the figure coincides well with the boundary of the dynamic stability region from Pai and Patton (1997), which suggests that the dynamic states that cause an instability of the apparatus would also cause an instability of a person. In Figure 6, the solid traces show the cases for which the rocking block recovered from the initial conditions, while the dashed traces show the cases for which the block fell. The block angle as a function of time corresponding to each case in the phase plane plot is also shown in the bottom plot of the figure.

Figure 7 shows the corresponding results for the rocking block experiments when using CGT assistance, controlled by the CGT controller described herein. Specifically, Figure 7 shows the angle and angular velocity trajectories corresponding to each trial with CGT assistance, where all angle and angular velocity data were measured using the Vicon motion capture system. The initial conditions corresponding to each trial are shown as *x*’s on the phase plot, while circles represent the instant at which thrust was triggered. As indicated in Figure 7, the block recovered from all initial conditions within the CGT-expanded stability boundary. One case with excessive initial velocity that is outside the expanded stability region resulted in a failed recovery. Note that for any case for which the angle and angular velocity of the apparatus remain within the limits of stability (within the bounds as indicated by the thick solid line in Figure 6), the CGT will not be triggered. As such, any perturbations from which the system can recover without the CGT, such as all traces indicated by solid lines in Figure 6, were not shown in Figure 7, since they would look exactly as they do in Figure 6 (i.e., will return to equilibrium without CGT assistance). Such cases would otherwise obfuscate the active responses shown in Figure 7.

The block angle as a function of time corresponding to each case in the phase plane plot is also shown in the bottom plot of the figure. Circles shown on the time plot similarly indicate the instant at which the controller triggered CGT thrust. The trajectories in the time plot were shifted to align the CGT trigger events at *t* = 0 to more clearly demonstrate the settling dynamics of the block. A video showing several of the trials without CGT assistance and with CGT assistance is included with the Supplementary Material accompanying this article.

As shown in the comparison between Figures 6 and 7, and also conveyed in Supplementary Video, the CGT was able to substantially increase the effective stability basin of the human-scale rocking block. Specifically, the CGT enabled the rocking block to recover upright equilibrium when subject to dynamic initial conditions that would otherwise have resulted in a fall. This data indicates substantial promise for CGT and the control approach proposed here.

### 5.1. Validity of single-segment model

Despite promising indications, this preliminary work entails a number of limitations. Among these, the controller is based on a reduced-order model of a falling human (i.e., a rocking block model), as was the experimental apparatus employed herein. As such, an important question is to what extent does such a reduced-order model approximate a falling human, at least in the portion of the phase space in which the CGT operates (i.e., the portion contained within the CGT-expanded boundary depicted in Figures 6 and 7). In particular, a human has multiple segments, while the rocking block model has one. There are at least two questions related to this: (1) to what extent does a rocking block describe a falling human; and (2) to what extent does a rocking block describe the response of the human to the imparted thrust? Regarding the first, a rocking block model is characterized by a rigid, single-DOF fall dynamic, while a human is a multi-segment entity that under large deviations will deviate from the dynamics of the rocking block model (i.e., sufficiently large joint angles will change the body moment of inertia and the length of the equivalent pendulum). As such, the rocking block model may not be sufficiently descriptive to provide effective control for a falling human. Regarding the second, a multi-segment body may respond to the thrust imparted by the CGT in a different manner than a rigid body (i.e., the body may “fold” in response to the thrust, which may prevent upright recovery). Although these questions cannot be fully resolved without extensive human-subject experimentation, the authors have attempted to inform these questions in the following sections of this discussion.

To help inform these questions, a multi-segment version of a falling human was simulated under different conditions and assumptions, with and without CGT assistance, and the relative responses were compared. In the multi-segment simulation, the human body was represented by a three-link pendulum consisting of a head-arms-trunk (HAT) segment, leg segment, and foot segment. The model was parameterized for a 1.55-m 52-kg person, in order to be consistent with parameterization of the experimental apparatus employed herein. The lengths, masses, and moments of inertia of the links were selected based on the average human body proportions and mass distributions described in Winter (2009). The ankle and hip joint torques were controlled using PD controllers that regulated desired joint velocity to zero and desired joint angle to a pose as described below, with joint torques saturated to align with reported joint torque limits for elderly individuals – specifically, 22 N m per leg (44 N m total) for the ankles (Thelen et al., 1996) and 51 N m per leg (102 N m total) for the hips (Cahalan et al., 1989). The ankle controller was simulated to regulate the COM angle to zero, while the hip controller was simulated for three different regulation strategies: (1) a “straight body” strategy, which is the closest configuration to the rocking block, where the trunk axis was aligned with leg axis; (2) a “vertical trunk” pose, where the trunk axis was aligned with the gravity vector; and (3) a “hunched forward” pose, where the hip joints remained fixed at a nonzero angle (40° in this simulation study), such that the trunk axis was offset from the leg axis. The 40° angle was chosen to fit a real-world scenario backward fall of an elderly person from the video described in the next subsection. The latter two control strategies were selected based on observations made in a study of recorded falls in elderly care facilities (Robinovitch et al., 2013; Choi et al., 2015). Note that all simulations were conducted using the OpenSim software environment (Delp et al., 2007; Seth et al., 2018).

For each hip control strategy, the response of the multi-link human was simulated for six different initial conditions, spanning the set of initial conditions performed in the rocking block experiments. Further, for each hip control strategy and initial condition set, the multi-link human was simulated with and without the CGT and control system, where the control system was simulated exactly as employed in the experiments (i.e., assuming a rocking block dynamic model, parameterized for a 1.55-m 52-kg person). As such, the controller is assumed to have real-time knowledge of the COM angle and angular velocity, and the system is also assumed to have a nozzle angle oriented at 90° relative to the COM axis. It should be noted that measurement of the COM angle and angular velocity would likely require IMUs mounted on the legs, which may or may not be realistic. Further, orienting the nozzle angle 90° relative to the COM angle requires the nozzle angle to be controlled prior to the initiation of thrust; further, technically, the nozzle should similarly be controlled following the initiation of thrust, although the change in angle during the thrust event should be insignificant. Therefore, the only assumption regarding nozzle control is that it be served prior to the thrust event to remain perpendicular to the COM angle.

**Figure 8.**
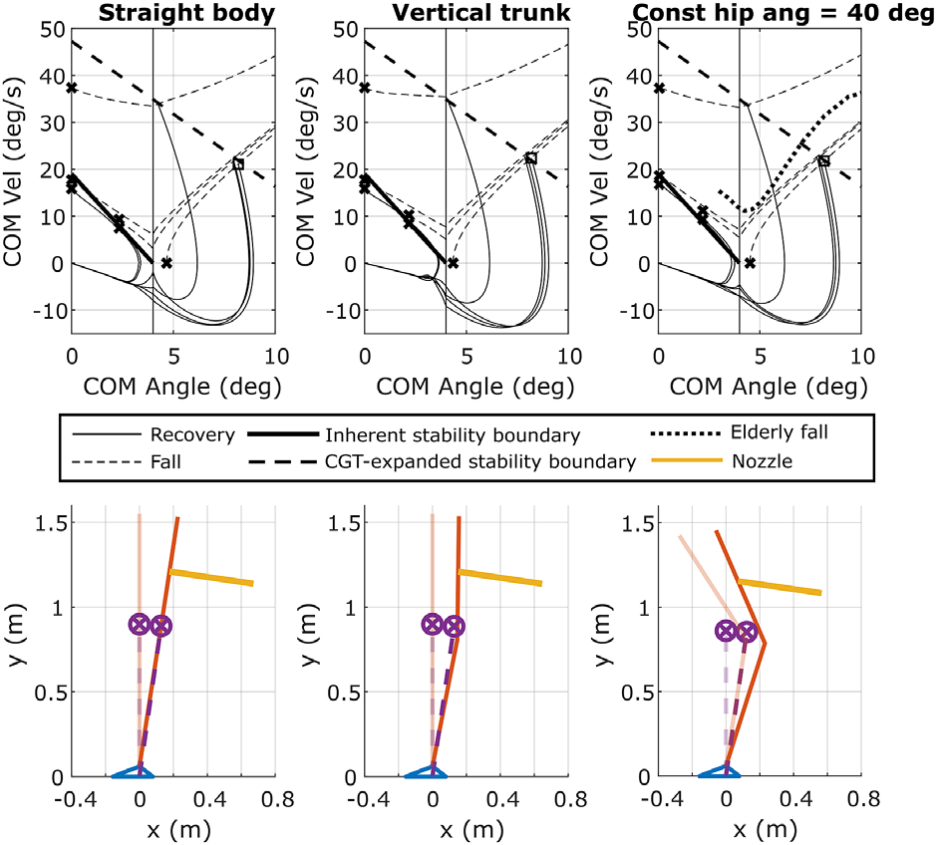
Results of simulation of a two-link pendulum model of a human falling backward. The top row displays the state evolutions of the COM from different initial conditions. Solid traces represent the cases where the pendulum returned to equilibrium, while the dashed traces show the cases resulting in a fall. The bottom row depicts the poses of the modeled human, where (a) trunk is aligned with legs, (b) trunk is aligned with the ground vertical, and (c) trunk is kept constantly offset from legs. The semitransparent human model is in the upright equilibrium, while the saturated human has the states corresponding to the square in the phase plot in the top row. The nozzle in orange is oriented perpendicularly to the COM axis.

Given these assumptions, a total of 36 simulations were conducted (i.e., three hip control strategies with six initial condition sets, each for the case with and without the CGT assistance). The results of these 36 simulations are depicted in Figure 8. Specifically, the top row of Figure 8 shows the phase plane trajectory for each case, while the bottom row shows the poses of the multi-segment human, in the equilibrium state, and at the instant the CGT is triggered. Note that the location and direction of CGT thrust (always perpendicular to the COM axis) are depicted in the figure with an orange line. In each phase plane, the backward LOS of 4° is denoted by the thin vertical line, to the left of which the model can reduce the backward velocity with ankle dorsiflexors, while to the right of it, the model has no means of preventing the fall. In each plot, an “*x*” denotes an initial condition; a solid trajectory shows recovery (no assistance required or after assistance was applied); and a dashed trajectory shows a fall. Note that a square in the plot indicates the instant at which the CGT is triggered (shown only for one case). As shown in the figure, the CGT controller effectively returns the simulated human to upright stability, regardless of the hip control strategy, despite the fact that the controller assumes a rigid posture. These series of simulations, despite the simplifying assumptions, indicate that the CGT controller, which is based on a reduced-order “rigid body” approximation of backward fall dynamics, may be robust to multi-segment joint movement, at least within the range of movement simulated here.

### 5.2. A case-study comparison with a real fall

**Figure 9.**
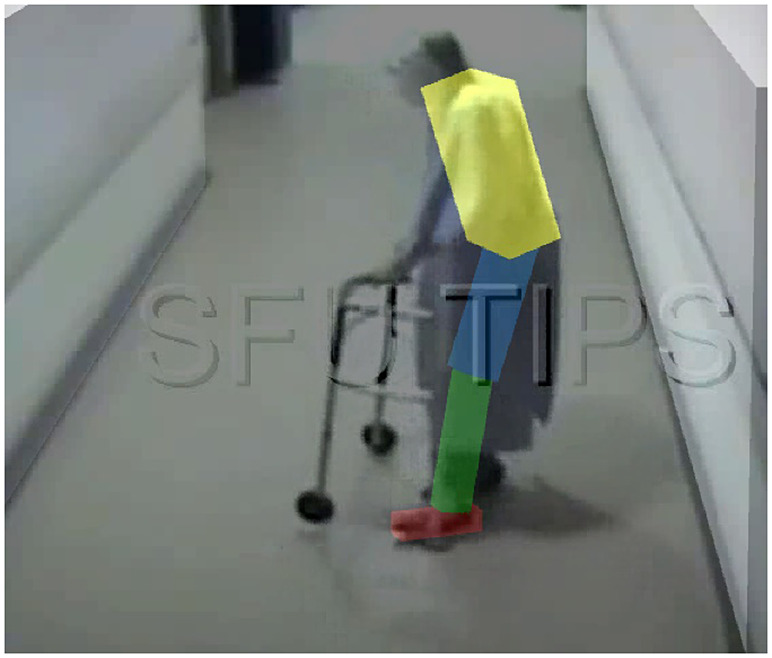
A frame from a video (available as a Supplementary Video at Robinovitch et al. (2013)) recording of a resident of an elderly care facility during a backward fall with the 3D matching procedure overlayed.

Ideally, the fall dynamics depicted in Figures 6– 8 would be compared to fall data recorded from “real-world” falls, which would offer a reference for the validity of the experiments and simulations. Such fall data, however, is not available (at least to the authors’ knowledge). In order to provide at least an approximate comparison, the authors extracted fall data from a video of a backward fall recorded in an elderly care facility, published by Robinovitch et al. (2013). A frame of this video is shown in Figure 9. In order to extract data regarding fall dynamics, the authors employed image processing software (fSpy, Stuffmatic) to extract the focal length and the spatial position of the camera used to record the video, and the extracted information was used to reconstruct the scene in space using open-source 3D modeling software (Blender Foundation). The 3D model frames were then imported into a 3D environment (Unity 3D, Unity Technologies), and the falling person’s silhouette was tracked in 3D space as a four-segment (foot, shank, thigh, and HAT) object, as shown in Figure 9. The positions and orientations of the segments, along with the nominal segment mass distribution from Winter (2009), were used to calculate the COM angle in the sagittal plane, which was then differentiated in MATLAB to obtain the COM angular velocity. The hip angle in this video was estimated as 40°. The state evolution of the falling person from the video is plotted and superimposed onto Figures 6 and 8 as a dotted line. Although the video subject attempts to use the walker to break the fall and their mass is unknown, the state evolution (as shown in time and on the phase-plane plots) is similar to that of the simulation (shown in Figure 8) and also of the rocking block experiments (shown in Figure 6), which further supports the conjecture that a reduced-order (i.e., rocking block) dynamics might be a valid approximation for the dynamics of the backward falling human body, at least within the phase-plane regime of relevance for CGT assistance.

## 6. Discussion

The results presented here indicate some promise as a means of arresting backward falls for people at fall risk. Several additional limitations exist. The prototype as presented considers backward falls; forward and sideways falls are also common. Addressing sideways falls would either require addition nozzles, or additional degrees-of-freedom in nozzle control. Addressing a forward fall might be more challenging, since a backpack-worn system cannot easily generate a backward thrust without interference from the body. Further, as mentioned in the simulation study, the controller as currently implemented requires measurement of the COM angle and angular velocity; for a multi-link system, such measurement would likely entail multiple IMU sensors, potentially one on each lower limb segment. Finally, while the mass of the system is fairly low (the current prototype is 2 kg, and a revised prototype could be made lighter), the thrust event is quite loud. Pneumatic silencers could potentially be employed to substantially reduce the sound level associated with the thrust event, although it is not clear to what extent employing such silencers would reduce the amount of corrective thrust. Therefore, although the proposed approach demonstrates promise, continued work is required to address these and associated limitations.

## 7. Conclusion

This article explores the use of a CGT, intended as a backpack-worn device, as a potential means of arresting backward falls for individuals at fall risk. While a previous paper examined the ability of the system to “restore” upright equilibrium to a statically leaning rocking block, this article described a controller that considers dynamic falls that are characterized by arbitrary combinations of center-of-mass angle and angular velocity. For such arbitrary conditions, the controller both detects impending falls and determines when the CGT should be energized in order to arrest the fall and restore upright equilibrium. The feasibility of the device and efficacy of the control approach were tested on a rocking block experimental apparatus, where the rocking block serves as a reduced-order model of a backward-falling human. In these experiments, the CGT and controller were shown to be highly effective, and in particular to expand substantially the stability basin of the block. In order to better understand the extent to which the reduced-order model employed in the control system and experiments might approximate a multi-segment falling human, various simulations were performed assuming a multi-segment human model assisted with the CGT controlled using the reduced-order (i.e., rocking block) controller. The simulations indicated that the controller based on a reduced-order model was able to effectively restore balance to the simulated multi-segment user under a variety of postural control assumptions. Therefore, despite several limitations associated with simplifying assumptions, the simulations indicate that the proposed control approach may be sufficiently effective when applied to a multi-segment human, at least within the regime in which the CGT is likely to be used. The authors plan to test this assumption with human subject experiments in future work.

## Supporting information

Baimyshev et al. supplementary material Baimyshev et al. supplementary material

## Data Availability

The collected data and the code used to process and plot the data are available at the following link: https://github.com/xkhannx/CGT_data.git.

## Supplementary material

The supplementary material for this article can be found at https://doi.org/10.1017/wtc.2023.18.
